# Magnetic resonance imaging and clinical features of Mayer–Rokitansky–Küster–Hauser syndrome: A 10‐year review from a dedicated specialist centre

**DOI:** 10.1111/1471-0528.17928

**Published:** 2024-08-12

**Authors:** Nina Cooper, Maya Al‐Memar, Kristofer Linton‐Reid, Keith Edmonds, Gillian Rose, Nuala Dixon, Cillian McNamara, Christina Fotopoulou, Katherine Van Ree, Nishat Bharwani

**Affiliations:** ^1^ Department of Disorders for Reproductive Development, Queen Charlotte's & Chelsea Hospital Imperial College Healthcare NHS Trust London UK; ^2^ Department of Metabolism, Digestion and Reproduction, Faculty of Medicine Imperial College London London UK; ^3^ Department of Imaging Imperial College Healthcare NHS Trust London UK; ^4^ Department of Surgery and Cancer, Faculty of Medicine Imperial College London London United Kingdom

**Keywords:** cyclical pelvic pain, Mayer–Rokitansky–Küster–Hauser syndrome, remnants, uterine remnants

## Abstract

**Objective:**

To correlate the clinical history with imaging findings of women with Mayer–Rokitansky–Küster–Hauser (MRKH) syndrome.

**Design:**

Retrospective cohort study.

**Setting:**

A UK IOTA and ESGO‐certified tertiary referral centre for disorders of reproductive development.

**Population:**

All patients with a diagnosis of MRKH and who had undergone an MRI pelvis between 1 January 2011 and 31 April 2021 were included.

**Methods:**

MRI images were analysed by specialist gynaecological radiologists. Clinical data was extracted from an electronic patient record system. Statistical analysis was computed in R (version 4.1.2), R base stats package and ggstatsplot (v0.5.0).

**Main Outcome Measures:**

Clinical history and predefined imaging features.

**Results:**

One hundred and thirty‐four patients were included. Median age at MRI was 18 years (10–64 years). Half (48.2%) of women presenting had a history of pain, most often abdominal (84.6%) or vaginal (9.2%). Remnants were identified in 91.8% of women (*n* = 123). 4.5% of women had imaging features of endometriosis (*n* = 6). Women with a functional remnants were significantly more likely to experience pain (*p* < 0.001). Pain history was not strongly associated with ectopic ovarian position. Common gynaecological pathology such as endometriosis, ovarian cysts and fibroids were also identified.

**Conclusions:**

We identify that majority of women with MRKH will have uterine remnants with a connecting fibrous band, and an ectopic ovarian position 44.0% of cases. Abdominal pain was significantly associated with functional remnants on MRI. Further work is required to identify how other gynaecological pathology impacts women with MRKH.

## 1 INTRODUCTION

### 1.1 Background

Mayer–Rokitansky–Küster–Hauser Syndrome (MRKH) is a congenital anomaly in the embryonic development of the genital tract, presenting with primary amenorrhoea in the presence of phenotypically female secondary sexual characteristics.^1^, ^2^ Uterine and vaginal agenesis occurs with resultant under‐developed rudimentary uteri (anlage, also called uterine remnants). Such remnants may have continuous fallopian tubes and can be cavitated with functional endometrial tissue. Uterovaginal hypoplasia may be isolated (type 1 MRKH), or associated with extragenital manifestations (type 2 MRKH). Most common are renal and skeletal anomalies.^3^ Affected women, and those assigned female at birth, have a 46XX karyotype, with 10% having detectable genetic micro‐deletions or micro‐duplications.^4^

Magnetic resonance imaging (MRI) is considered the gold standard for MRKH, however, ultrasound (US) is the first‐line imaging modality for the investigation of the female pelvis.^4^, ^5^ Uterine remnants or ectopic ovaries may not be pictured with adequate certainty on US.^4^ US therefore has an important screening role in identifying the presence of female internal genital to exclude MRKH. In the hands of an advanced operator, US can offer a comprehensive anatomical assessment in those with the condition and may be appropriate in settings where MRI is inaccessible.^6^ Practical issues must be considered as women with MRKH may only have a vaginal dimple, rendering transvaginal US impossible and transrectal US has a lower sensitivity in the detection of uterine remnants.^4^, ^7^

MRI offers a more sensitive, non‐invasive method for assessing pelvic anatomy. MRI identifies uterine remnants in most patients,^8^, ^9^ demonstrating anything from a single‐layered structure to a three‐layered uterine remnants with functioning endometrium (functional remnants).^5^ Functional endometrium is associated with symptoms such as pelvic pain and complications associated with retrograde menstruation, such as endometriosis.^10^

Most studies evaluate the utility of MRI as correlated with surgical findings, not clinical symptoms.^7^, ^8^, ^9^, ^11^, ^12^, ^13^ Data on specific MRI features and their correlation with clinical symptoms in guiding management is limited. We hypothesise that the imaging features of MRKH could correlate with clinical symptoms, specifically that the presence of functional tissue within a remnant could be associated with cyclical symptoms and gynaecological pathology such as endometriosis. This could provide a framework on which to offer targeted onward management.

### 1.2 Objectives

Our primary aim was to review the MRI features of MRKH. The secondary aim was to correlate the imaging findings with clinical history, with a view to guiding management. The null hypothesis was that MRI would not correlate with the clinical features of MRKH.

## 2 METHODS

This retrospective review of clinical data and imaging was approved by our institutional review board, with a waiver for informed consent. A core outcome set was not used.

### 2.1 Inclusion criteria

**FIGURE 1:**
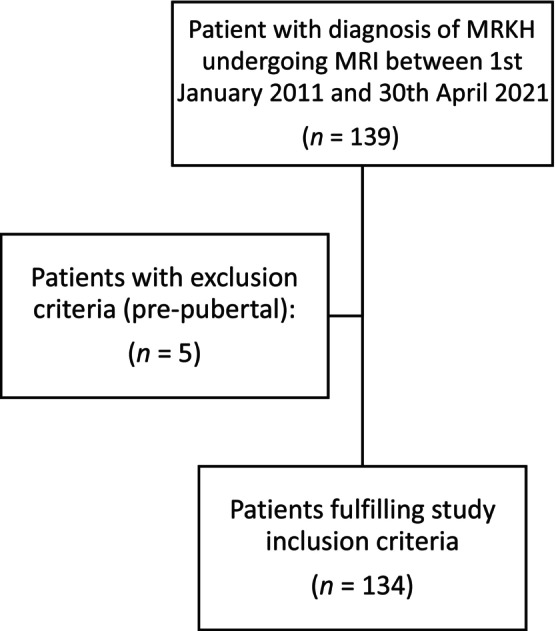
Flowchart detailing the number of patients who fulfilled the study criteria.

Patients who were referred to the specialist clinic in a tertiary hospital with a diagnosis of MRKH or seen within this clinic and diagnosed with MRKH, and had undergone an MRI pelvis between 1 January 2011 and 31 April 2021 were included (Figure 1). Patients were identified from a weekly MDT record kept by the institution and clinical coding. Patients were excluded if they were pre‐pubertal at the time of imaging. Development of secondary sexual characteristics was used as a surrogate marker of puberty due to absence of menses within this group.

### 2.2 MR imaging

MRI studies were either performed locally or images were imported from other centres. Those performed at our centre used either a Siemens Magneton Aera 1.5T or a Philips Achieva 1.5T scanner, both employing a body‐array coil. The protocol used is summarised in the [Link], [Link], [Link]. To improve image quality, all patients were administered hyoscine butylbromide (Buscopan) 20 mg/mL via intramuscular injection prior to imaging unless contraindicated.

The large field of view (FOV) axial T2W images were acquired from the symphysis pubis to the renal hila. The axial T1W sequences (without and with fat saturation), and small FOV axial, coronal and sagittal T2W sequences covered the pelvic anatomy only.

### 2.3 Outcomes

Primary outcomes were the presence or absence of predefined imaging characteristics. Secondary outcomes were predefined clinical characteristics and correlation between clinical features and imaging characteristics.

### 2.4 Image analysis

Images were reported by specialist gynaecological radiologists and imaging data were extracted from an electronic picture archiving and communication system (Carestream PACS®). For each MRI the following information was extracted: (a) presence or absence of uterine remnants, (b) presence of endometrial tissue, (c) presence and position of ovaries, (d) vaginal length, (e) visible fibrous band, (f) any associated anomalies (Figures [Link], [Link]). An ectopic ovary was defined as an abnormally located ovary above the level of the pelvic brim (the pubic symphysis to the sacral promontory line) or located far laterally just behind the anterior abdominal wall.^14^ Uterine remnant volume was calculated using the formula for an ellipsoid (volume = 4/3 × *π ABC*). Normal vaginal length was defined as 7–9 cm. A fibrous band was visualised as a V‐shaped line of soft tissue lying above the bladder dome in the expected position of the uterine cervix.

Where data were missing from the formal report, specialist radiologists subsequently reviewed images flagged by the principal investigator.

### 2.5 Clinical data

Patient demographics and clinical outcomes were extracted from an electronic patient record system (Cerner®). A proforma was created to standardised clinical data extraction. As extraction was retrospective, no standardised pain scale could be used. Recorded clinical outcomes included: age at diagnosis, presentation to clinic and MRI, pain history, medical treatments for pain, presence of gynaecological pathology and relevant surgical findings (Table 1).

**TABLE 1:** Patient demographics including age at MRI, age at referral to our specialist service, and self‐declared ethnicity. Details of the clinical history including presence of pain history, site and pattern of pain and use of medication for symptomatic relief.

	Median	Range
Age at MRI (years)	18.00	10–64
Age at referral (years)	19.00	10–64
	***N***	**%**
Ethnicity		
White	62	46.2
Asian	14	10.4
Black	1	0.8
Mixed	1	0.8
Other	51	38.1
Not documented	5	3.7
Pain history		
Yes	65	48.2
No	66	49.6
No data	3	2.2
Site of pain		
Abdominal	55	84.6
Vaginal	6	9.2
Other	4	6.02
Pattern of pain		
Cyclical	30	22.4
Variable	28	20.9
Provoked	8	5.9
No data	68	50.7
Medication use		
None	5	3.7
OCP	5	3.7
Analgesics	3	2.2
No data	121	90.3

### 2.6 Analysis of data

Comparisons between features are summarised using Spearman correlation between continuous features, and Fisher's exact test for between categorical features, and when comparing continuous variables between groups *t*‐test was used (for two groups) or ANOVA (three or more groups). Statistics were computed in R (version 4.1.2), R base stats package and ggstatsplot (version 0.5.0) and IBM SPSS version 29. Analysis of pain history was prospectively planned and therefore no retrospective subgroup analysis was performed.

## 3 RESULTS

One hundred and thirty‐four patients fulfilled the study inclusion criteria. Five were excluded as pre‐pubertal (Figure 1). Fifty MRI scans were performed internally, and 84 were performed externally. The median age at MRI was 18 years (10–64 years). Median age at referral to our centre was 19 years (10–64 years) (Table 1). Ethnicity was self‐reported: 46.2% White, 10.4% Asian, 0.8% Black, 0.8% mixed ethnicity, 38.1% ‘Other’ and 3.7% had no ethnicity recorded. Clinical history features are described in Table 1. 48.2% of women had a positive pain history, and of these women, this was most commonly abdominal pain (84.6%).

Forty‐five patients had an associated renal and/or skeletal anomaly, meaning 65.1% had type 1 MRKH (isolated uterovaginal hypoplasia) and 34.9% had type 2 MRKH. Ectopic position of uterine remnants was not associated with a positive pain history (*p* = 0.07 for right‐sided remnants, *p* = 0.99 for left‐sided remnants). The presence of a functional remnant was significantly associated with the presence of pain (*p* < 0.001). Women with functional remnants were significantly more likely to receive medical treatment for their pain (*p* = 0.03). There was no significant association between size of remnant and positive pain history in both right‐sided and left‐sided remnants. 48.4% of women with cyclical pain (15/31) and 26.9% of women with variable pain (7/26) had visible endometrium on MRI.

### 3.1 Uterine remnants and endometrium

A summary of the descriptive MRI findings can be seen in Table 2 and detailed statistical analysis is available as Figure S1. Uterine remnants were present in 91.8% of patients (*n* = 123). Where present, remnants were bilateral in 94.3% of cases (*n* = 116) and unilateral in 6.7% (*n* = 9). The remnant was distant from the ipsilateral ovary in three cases; otherwise, the remnant was identified caudal to the ipsilateral ovary regardless of the anatomical position of the ovary. A fibrous band was present in 94.0% (*n* = 126). Table 2 demonstrates the MRI characteristics seen within this cohort.

**TABLE 2:** MRI findings in women with MRKH.

	Yes		No		Indeterminate	
	*N*	%	*N*	%	*N*	%
Ovaries visualised	129	96.2	4	3.0	1	0.7
Remnant visualised	123	91.8	9	6.7	2	1.5
Fibrous band visualised	126	94.0	8	5.9	0	0
Endometrium present						
Right	18	13.4	97	72.4	3	2.2
Left	22	16.4	103	76.9	1	0.7
Renal anomaly	36	26.9	72	53.7	19	14.2
Skeletal anomaly	18	13.4	116	86.6	0	0
Endometriosis	6	4.8	124	92.5	4	3.0

	Right		Left	
	*n*	%	*n*	%
Remnant present				
Yes	116	85.93	119	88.15
No	12	8.89	9	6.67
Indeterminate	6	4.44	6	4.44
Remnant position				
Normal	114	93.4	119	95.2
Ectopic	5	4.1	4	3.2
Inadequately visualised	3	2.5	2	1.6
Ovary position				
Normal	99	73.3	96	72.7
Ectopic	30	22.2	36	27.3
Ovarian appearance				
Normal	124	96.1	120	90.9
Abnormal	5	3.9	10	7.6
Indeterminate	0	0	2	1.5
Mean remnant volume (mL)	23.9		26.6	
Mean endometrial thickness (mm)	5.8		5.0	

Functional endometrium was demonstrated within the uterine remnants in 31 patients. Endometrial tissue was present bilaterally in 12 patients, renal and skeletal anomalies were only present in one of these cases. Mean endometrial thickness was 5.8 mm in right remnants and 5.0 mm in left remnants, ranging from 1.8 to 12.0 mm. Mean remnant volume was 23.9 mL on the right and 26.6 mL on the left. Larger remnants were significantly more likely to be functional (*p* = 0.01 for right‐sided remnants; *p* = 0.03 for left‐sided remnants). There was an association between remnant volume and endometrial thickness, but this was not consistently statistically significant (*p* = 0.03 right‐sided remnants, *p* = 0.06 left‐sided remnants).

Endometriosis was observed in six cases and in two of these patients, no endometrial tissue was identified in the uterine remnants. Five out of six of these women suffered from pelvic pain.

### 3.2 Ovaries

Ovaries were present in 96.2% (*n* = 129). One hundred and twenty‐eight of these 129 patients had bilateral ovaries (99.2%). Ovaries were not identified in four patients due to the limited field of view on MRI in externally imported studies, and one study was technically inadequate. Ectopic ovary position was noted in 22.4% on the right and 26.9% on the left. Ectopic ovarian position in at least one ovary was seen in 59 women (44.0%): 31.4% (*n* = 42) patients had a single ectopically positioned ovary whilst 17 had bilateral ectopic ovaries (12.7%). Ectopic ovarian positions are available in Table S2. Ovarian pathology was noted in 17 cases (12.6%), and findings are summarised in Table 3. The ovarian position was not significantly associated with a positive pain history. Ovarian pathology was also not significantly associated with a history of pain.

**TABLE 3:** Presence of ovarian pathology in women with MRKH.

Ovarian pathology	Right	Left	Total		% Overall (*n* = 134)
	*n*	*n*	*n*	%	
Simple cyst	1	1	2	11.8	1.49
Haemorrhagic cyst	2	3	5	29.4	3.73
Abnormal shape	2	2	4	23.5	2.99
Paraovarian cyst	1	1	2	11.8	1.49
Dermoid cyst	0	1	1	5.9	0.75
Atypically small	0	1	1	5.9	0.75
Suspected malignancy	0	1	1	5.9	0.75
Endometrioma	0	1	1	5.9	0.75
			17	100	12.69

### 3.3 Vagina

Vaginal length was within normal limits in one patient (8 cm) who had self‐dilated. In the remaining 133 patients (99.3%), varying degrees of vaginal hypoplasia were observed. Thirty‐eight patients had a vaginal dimple or no measurable vagina (28.4%). Overall median vaginal length, including those with vaginal dimple <0.5 cm in size, was 1.9 cm (0.0–8.0 cm). For those with a measurable vagina, mean vaginal length was 2.3 cm (0.5–8.0 cm). The seven women complaining of vaginal pain had a vaginal length varying from a dimple to 3.5 cm and all reported that this was provoked by intercourse. Women who complained of pain had an overall significantly greater vaginal length (2.4 cm vs. 1.6 cm, *p* = 0.04) than those who did not.

### 3.4 Associated anomalies

Renal anomalies were identified in 36 (26.9%) patients ranging from malrotation, and pelvic location of kidneys to complete absence of one kidney. Skeletal anomalies were visualised in 18 (13.4%) sacrococcygeal anomalies (*n* = 6) being the most frequently observed. Half (9/18) of the patients with a skeletal anomaly had an associated renal anomaly. Further information is available in Table S3. One of the patients with scoliosis also had bilateral dysplasia of the femoroacetabular joints and another had a single dysplastic hip.

## 4 DISCUSSION

### 4.1 Main findings

Although many series explore the imaging and clinical features of MRKH as separate entities, this is the largest analysis specifically correlating clinical features and imaging appearances of MRKH to date. Most women with MRKH have bilateral uterine remnants with a connecting visible ‘v‐shaped’ fibrous band and have bilateral ovaries, which are ectopically positioned in 44% of cases. Almost half of women with cyclical abdominal pain had visible endometrium within the uterine remnants on MRI.

Previous studies suggest that uterine remnants may be visible in 68%–100% of cases.^8^, ^9^ A review of 66 MR images identified uterine remnants in 93% of patients, which increased to 95% of cases upon laparoscopic review.^13^ Similarly, a study of 56 women confirmed MRI sensitivity of 81.42%, corresponding with laparoscopy findings (*k* = 0.55).^11^ In our study, uterine remnants were identified in a total of 91.8% of patients.

The volume measurements of the uterine remnants are similar to those described by Wang et al. (33.5 mL for unilateral remnants, 16.1 mL for bilateral remnants) however they were larger than those described by Hall‐Craggs et al. (mean volume = 6.4 mL).^8^, ^13^ The cause for this discrepancy is unclear but may be related to inherent differences in the cohorts. A fibrous band was observed in 94.0% of cases. Previous studies have observed this structure in 48%–90% of cases.^5^, ^15^

Almost half of our study population reported abdominal pain, compared to just 1.4% of a national study of 1055 patients.^16^ This may be due to differences in study methodology or reflect the heterogeneity of MRKH as a condition itself. In our population, visible endometrium was associated with a significantly higher incidence of any pain type compared to those without. Marsh et al. reported visible endometrium in 64% of patients with pelvic pain in MRKH.^17^ In our cohort, of those patients who experienced cyclical pelvic pain, a similar number (60.7%, 17/28) had endometrium visualised within their uterine remnant. This demonstrates the potential utility of MRI in guiding management. Where endometrium is not visualised, other causes of cyclical pain should be considered. Cyclical pain may be ovulatory, where hormonal therapy may still be appropriate and non‐cyclical causes such as bowel or bladder pathology should also be considered.

Theoretically, timing of MRI within the menstrual cycle may influence the visibility of endometrium. However, if there has been sufficient differentiation to the point of having endometrial tissue, it should always be visible Larger remnants had significantly greater endometrial thickness, which may allow for easier identification. Wang et al.^5^ demonstrated that most remnants have single‐layer differentiation of the endometrium, however, 15.6% had two‐ or three‐layer differentiation or haematometra or haematosalpinx. Furthermore, a 2024 study showed 40.8% of patients had endometrium on histological examination of remnants, with more cases of functional remnants seen in type 1 MRKH compared to type 2. Similarly, we identified that only one patient with bilateral functional remnants had an associated skeletal or renal abnormality. This may be due to the differences in the development of the remnants.^16^ Surgical removal of remnants is feasible, however the relative risks and the future impact of surgery must be considered. Specifically, a history of prior pelvic surgery is a current contraindication to uterine transplantation.

One patient in this study without any visible endometrium had a 6.4 × 4.3 cm rectovaginal structure possessing all the imaging features of an endometrioma. This suggests that those without MR‐detectable endometrium can still suffer from endometriosis and the associated symptoms. As far as the authors are aware, this is the first paper to describe such a finding. The most likely mechanism for this is retrograde menstruation. Remnants have been found to possess fallopian tubes; however, these were not specifically identified in this study.

The incidence of ectopic ovaries in the literature is variable ranging from 12.5% to 42%^7^, ^8^, ^12^, ^13^ which may be due to varying definitions of what is classified as ectopic. The majority of ectopic ovaries in our study were located either overlying psoas or present laterally or posteriorly to the large bowel. Two ovaries were visualised in the inguinal canal which has been previously reported.^18^ Overall, there was no association between the position of the ovary and pain history. The ovarian position is especially important for patients who wish to undergo oocyte retrieval, and a TA approach may be preferred with ectopic ovaries.^19^ Retrocaecal ovarian position was identified in 16.7% of right‐sided ovaries, whilst 8.3% of left ovaries were posterior to the descending colon in our cohort, which is important to note to avoid bowel injury.

9.2% of our study population complained of dyspareunia, which is similar to another study with a prevalence of 6.7%.^16^ Vaginal length was within normal limits in one patient (8 cm) due to prior dilator treatment. In the remaining patients (99.3%), varying degrees of vaginal hypoplasia were observed. The average vaginal length of 2.3 cm in our cohort is consistent with that described by Wang et al. (2.4 cm).^8^ All women who reported vaginal pain described provoked dyspareunia. Interestingly, women with a higher mean vaginal length reported greater pain overall (2.4 cm vs. 1.6 cm), the cause for this is not clear. Studies have demonstrated that this resolves with vaginal dilator treatment, resulting in similar overall sexual satisfaction.^20^, ^21^, ^22^

The overall prevalence of type 1 and type 2 MRKH (65.1% and 34.9%, respectively) was similar to that of a national study of 1055 patients (69.6% and 30.4%, respectively).^20^ Renal anomalies were identified in 36 (26.9%) of our study population, which closely aligns with a 2024 study by Pietzch et al. who identified anomalies in 22.8% of their population.^5^, ^7^, ^16^, ^19^, ^20^, ^21^ Skeletal anomalies were visualised in 13.4% of patients which is in keeping with findings by other groups who describe skeletal malformations in 15.1%–22.0% of cases.^16^, ^20^, ^23^, ^25^ We did not identify any cases of MURCS (Müllerian, Renal and Cervicothoracic somite abnormalities) or VACTERL (vertebral defects, anal atresia, cardiac defects, tracheoesophageal fistula/oesophageal atresia, renal anomalies, and limb abnormalities) in our population, which have been described in other series.^4^, ^20^, ^23^, ^24^

### 4.2 Strengths and limitations

The strengths of this study include the number of patients; this is the largest study of MRI images in MRKH to date. We are a national referral centre and therefore have expertise in the investigation and management of these patients. All images were reported by gynaecological radiologists with specific experience in this cohort, allowing for specific identification of the key features of MRKH. Furthermore, we have described not just the MRKH features, but are the first study to analyse the statistical relationship between MRI features and clinical history, with suggestions for how this can guide real clinical practice.

The limitations of this study are related to retrospective collection of data and therefore no standardised or quantitative pain scale could be used. Inconsistencies in documentation of the clinical history resulted in incomplete pain histories. Furthermore, MR studies were imported from referring hospitals and the exact MR protocol is therefore not controlled.

### 4.3 Interpretation

MRI presents a sensitive, non‐invasive and safe tool to guide onward management for women with MRKH who are experiencing gynaecological symptoms. This study also serves as a reminder that women with gynaecological symptoms and a diagnosis of MRKH can experience malignant and benign gynaecological pathology, which is not directly related to their condition.

## 5 CONCLUSION

Our study indicates that uterine remnants are present in the vast majority of patients with MRKH and are most often bilateral. A visible fibrous band can be seen connecting the uterine remnants in over 90% of cases. Ectopically positioned ovaries were not associated with symptoms of pain. Women with functional remnants on MRI were significantly more likely to experience pelvic pain. Therefore, MR imaging can guide further management, such as hormonal therapy or surgical excision of remnants, particularly where distension of the remnants with blood or shedded endometrium is thought to be the underlying source of pain. MRI also allows for the identification of other causes of pelvic pain such as endometriosis. As the incidence of abdominal pain is relatively high in this patient cohort, further studies are needed to understand the underlying aetiology.

## AUTHOR CONTRIBUTIONS

NC, MA‐M, CM and NB wrote the manuscript. NC, CM, KVR and NB collected data. NC and KL‐R cleaned and analysed the data. NB and KVR provided MR images. KE, GR, CF and ND contributed to the editing and content of the manuscript.

## FUNDING INFORMATION

There was no funding.

## CONFLICT OF INTEREST STATEMENT

The authors declare no conflict of interest.

## ETHICS APPROVAL

None.

## Supporting information


Figures S1–S3

Figure S4

Tables S1–S3

## Data Availability

Research data are not shared.
