# Supplementary material for: Magnetic resonance imaging and clinical features of Mayer–Rokitansky–Küster–Hauser syndrome: A 10‐year review from a dedicated specialist centre
Source: BJOG. 2024 Aug 12;132(1):64–71. doi: 10.1111/1471-0528.17928 (PMC11612609; doi:10.1111/1471-0528.17928)
Supplement: Supplementary file 1 — Figures S1–S3 [file BJO-132-64-s003.docx]

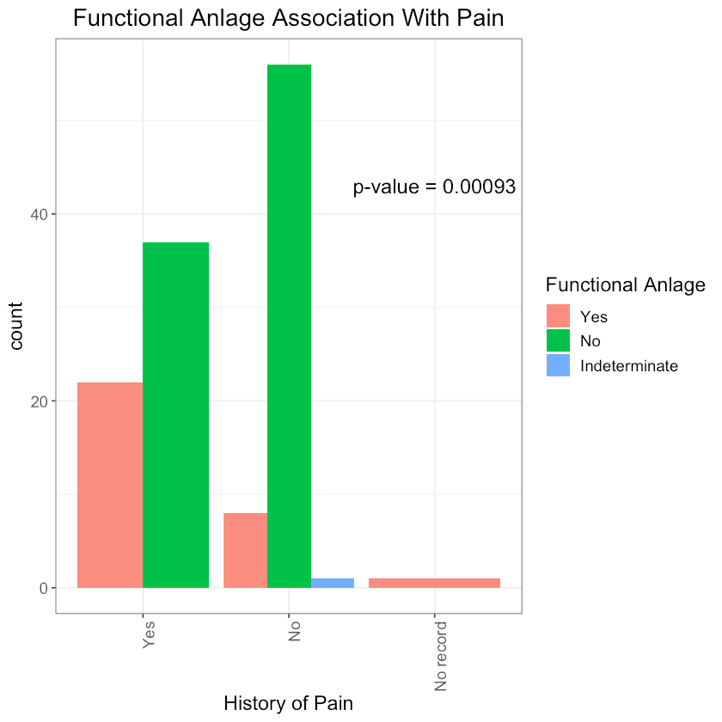

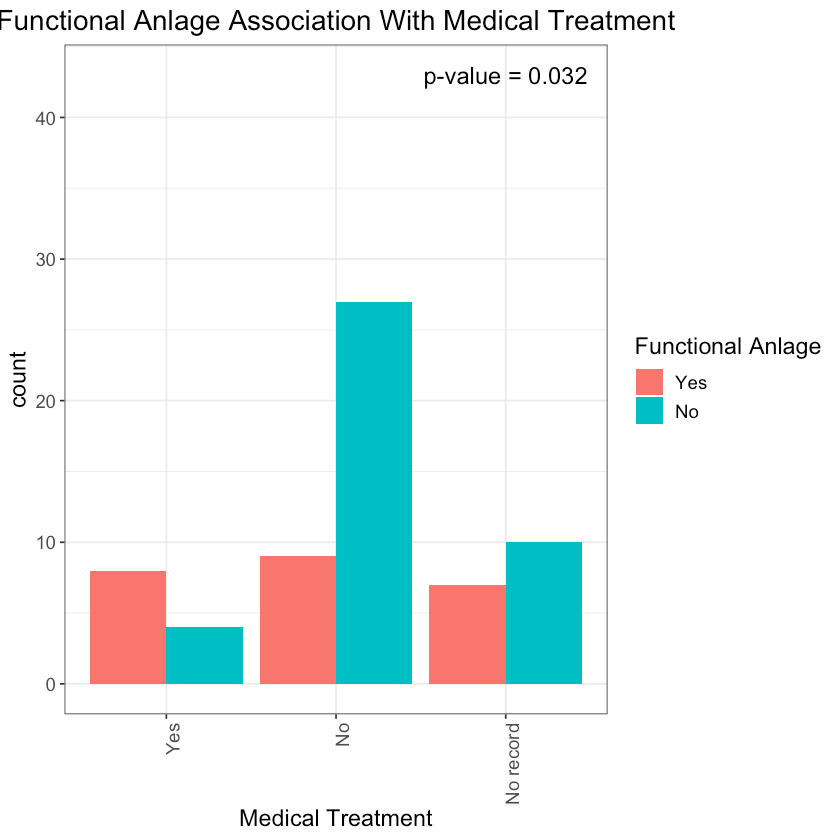

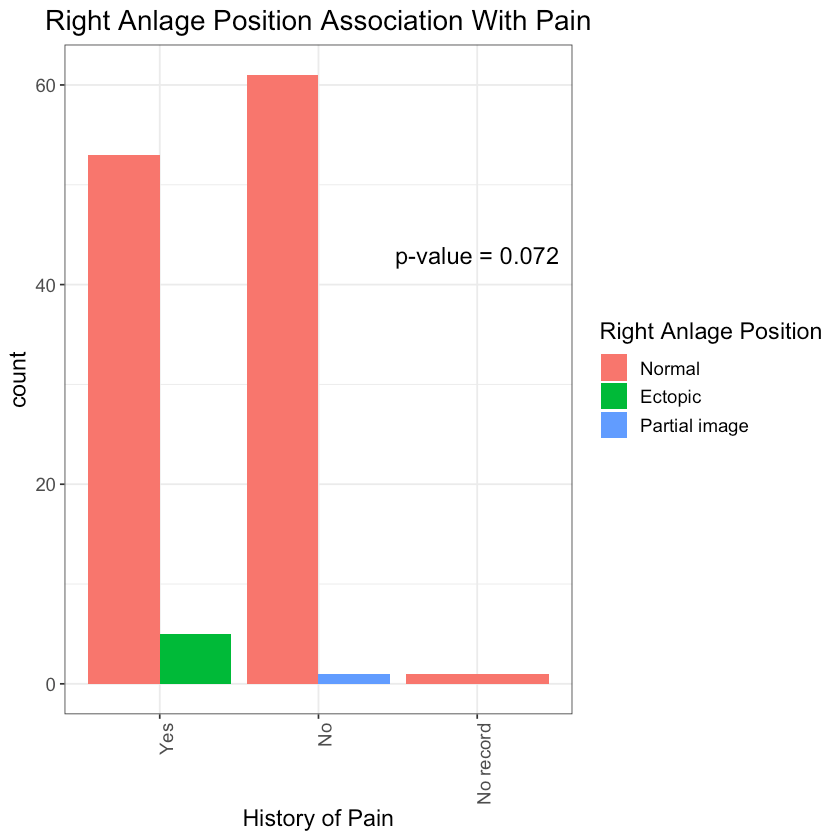

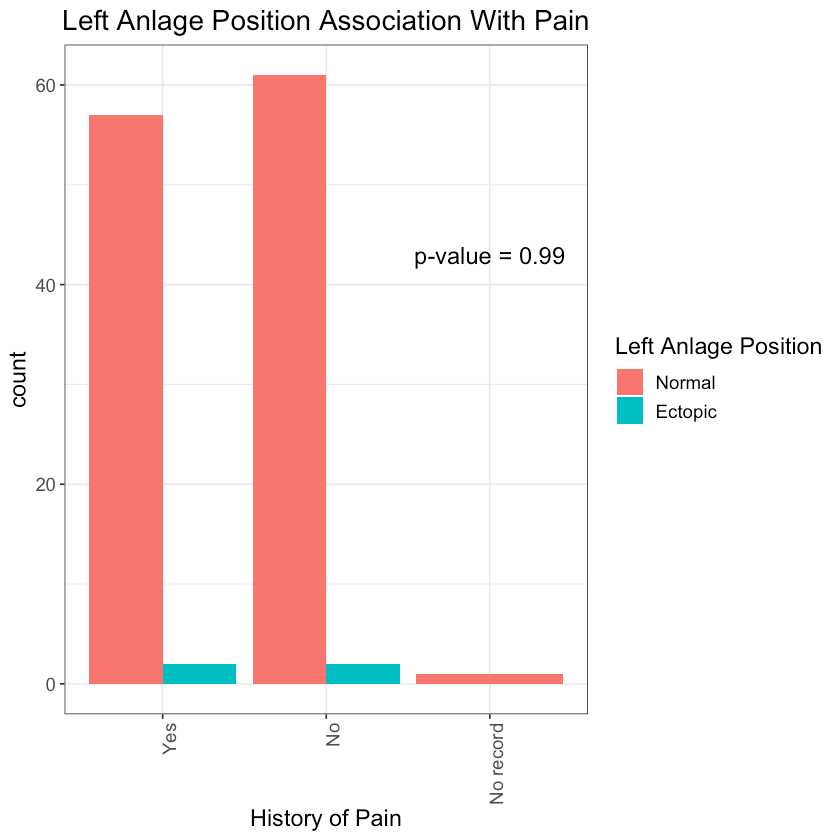


d

c

b

a


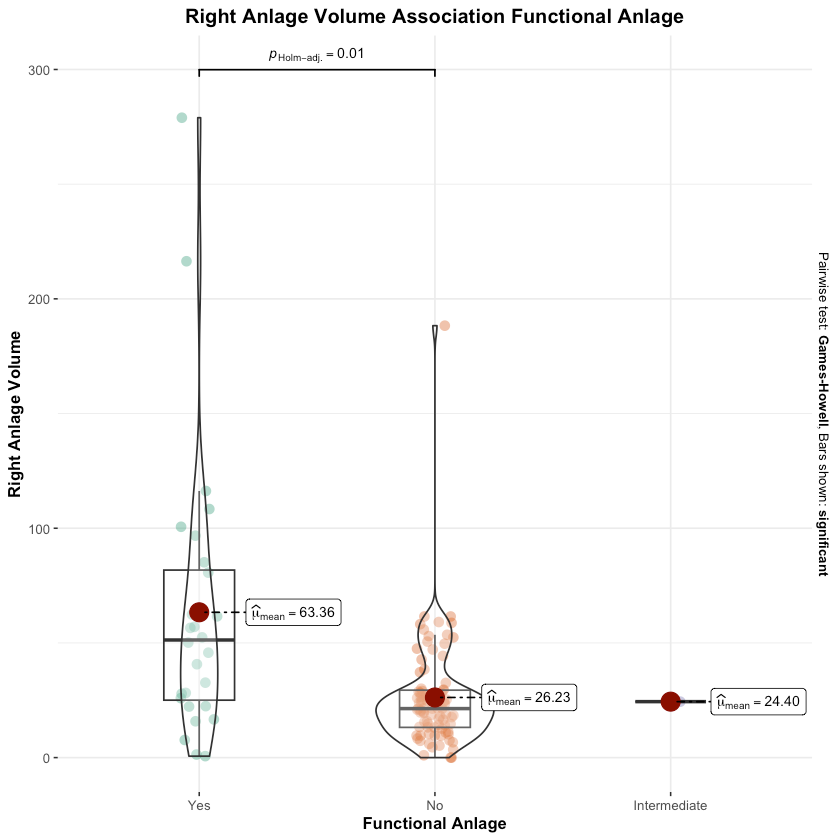


f

e


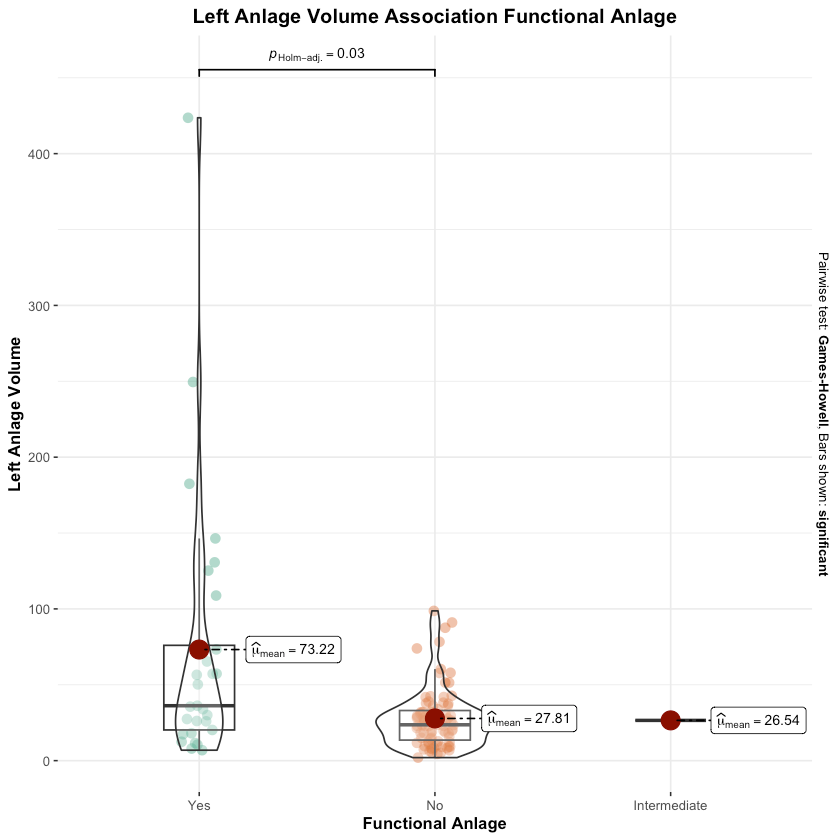


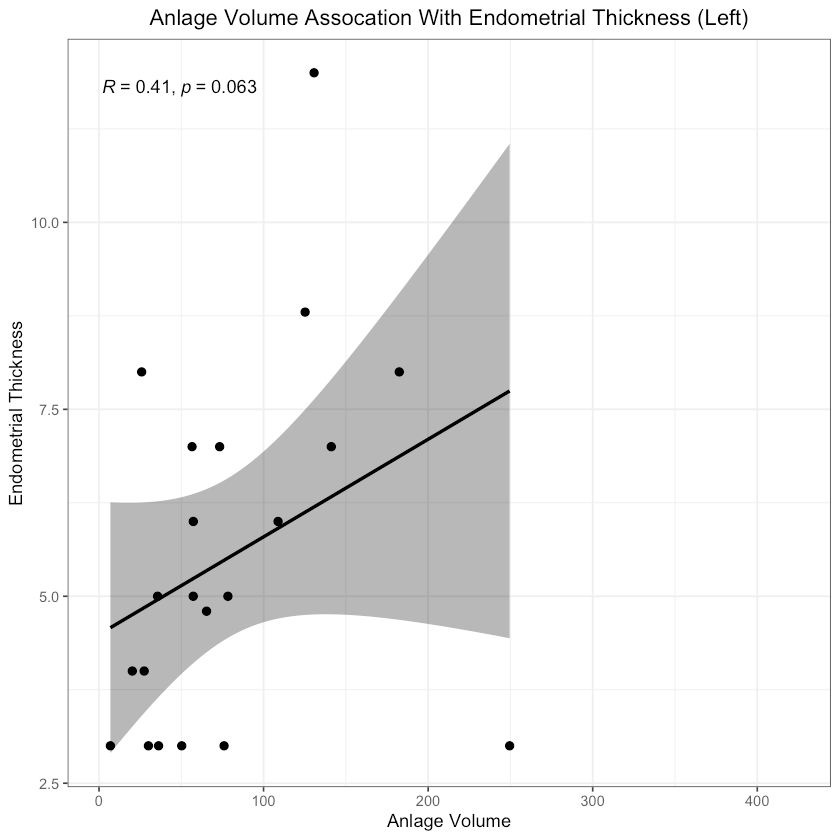

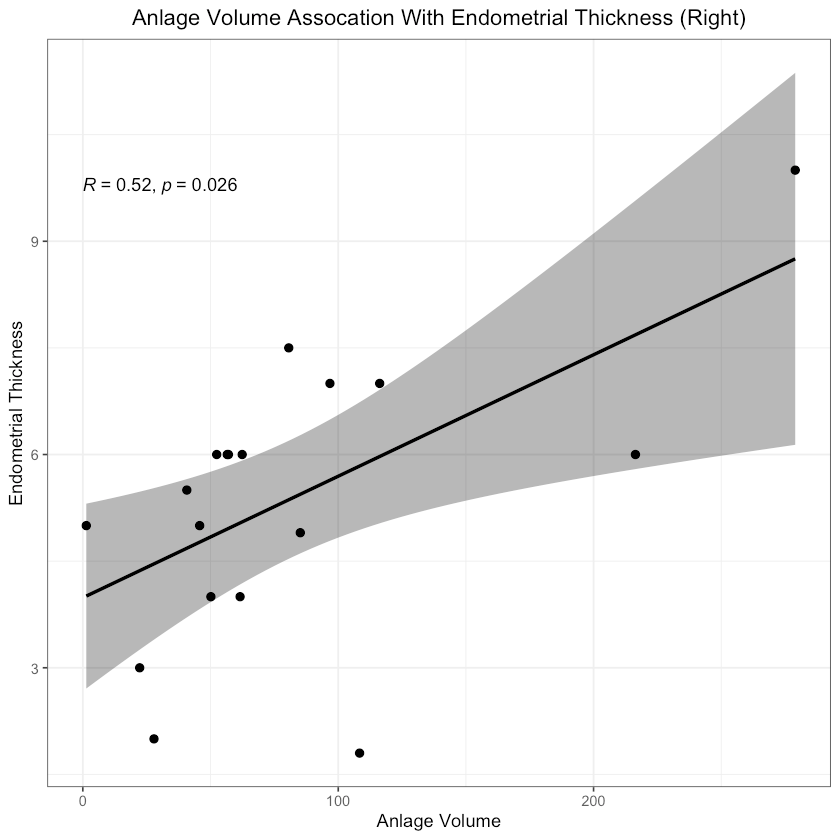


h

g


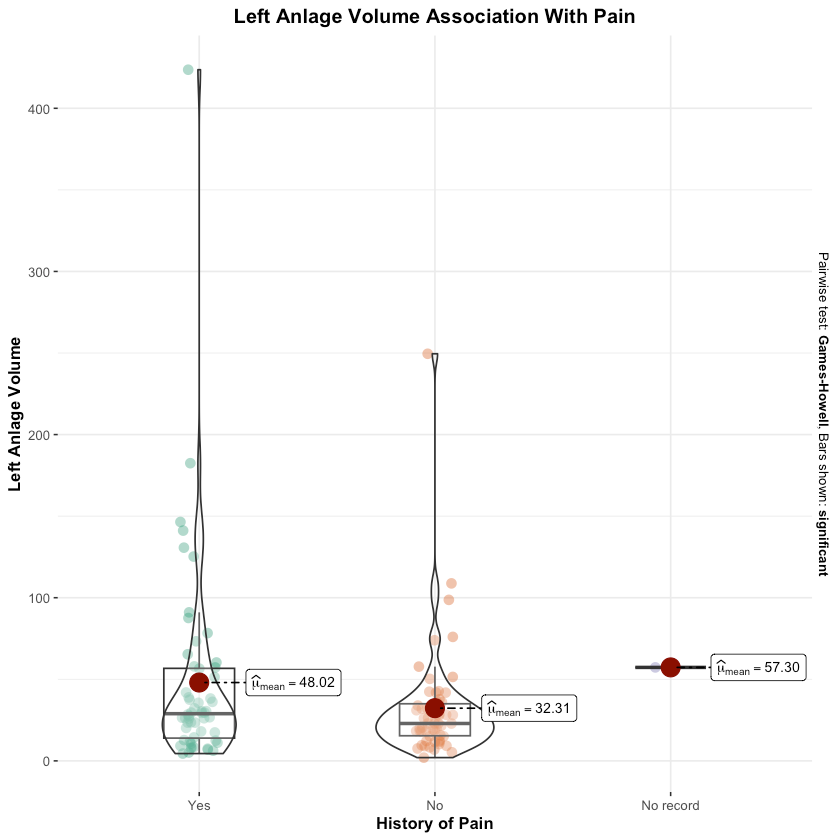

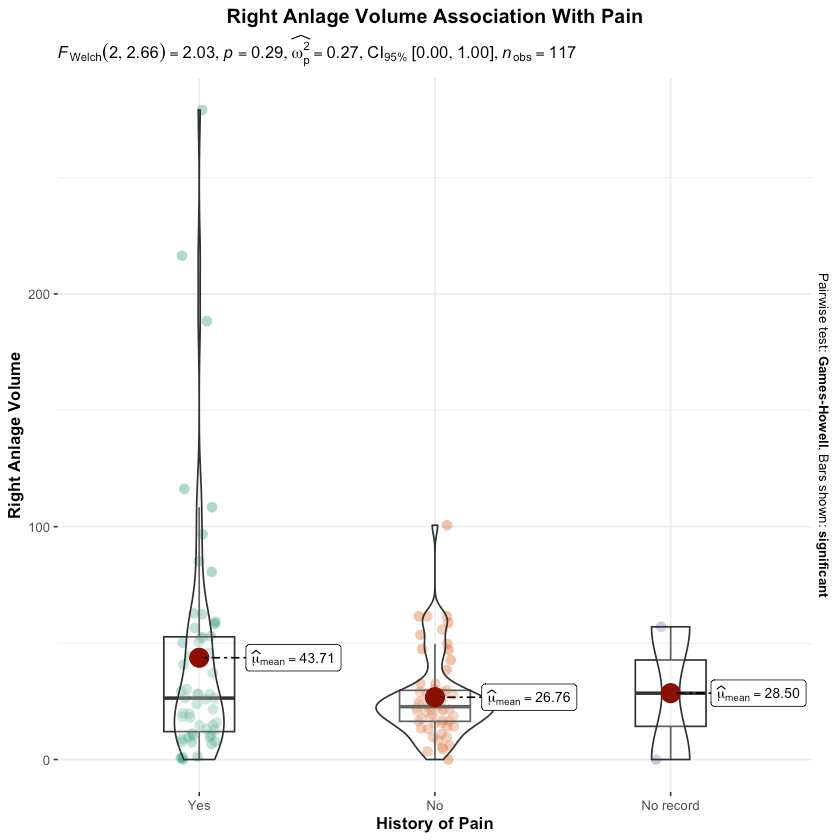


j

Figure S1: Anlage characteristics and clinical history

a, b: Relationship between pain history and position of anlage

c: Relationship between pain history and presence of functional anlage

d: Relationship between need for medical management of pain and presence of functional anlage

e,f: Relationship between volume of anlage and presence of functional endometrium

g,h: Relationship between volume of anlage and thickness of endometrium

i,j: Relationship between volume of anlage and pain history


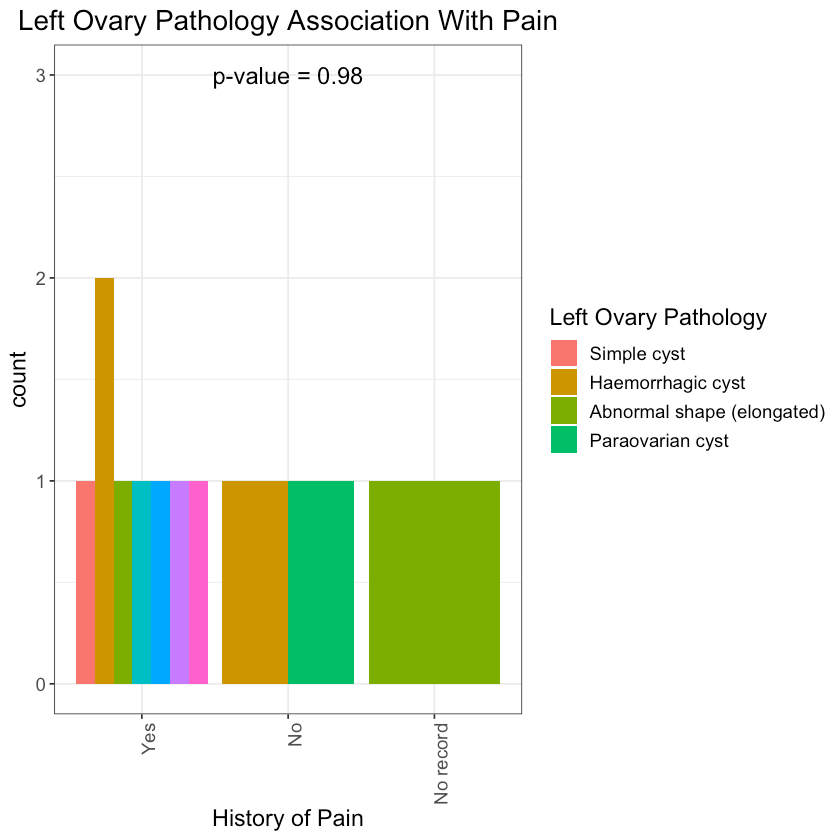

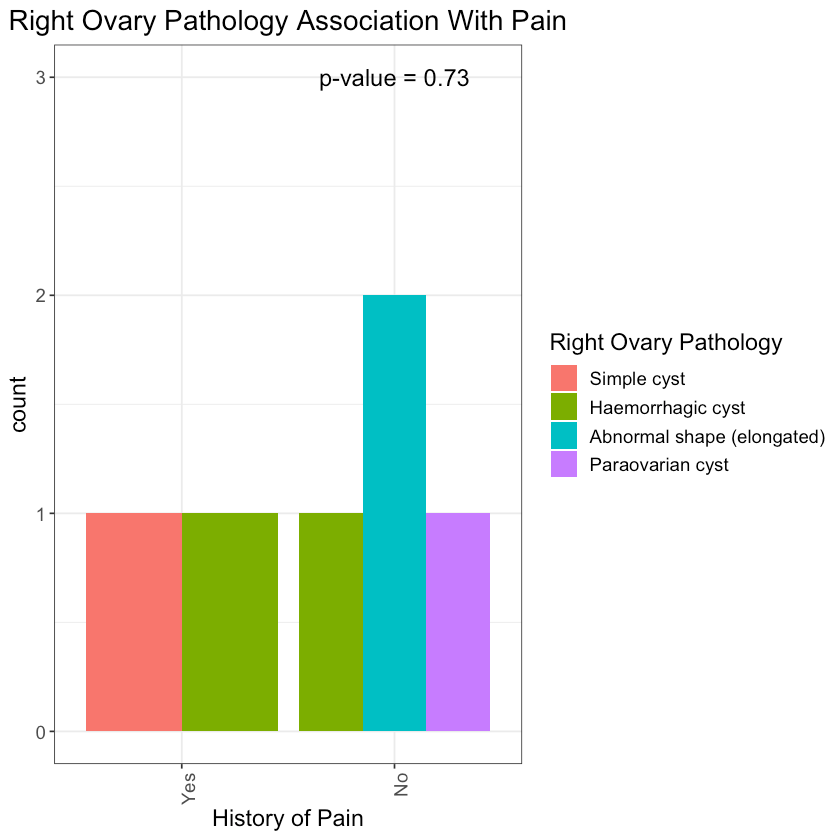

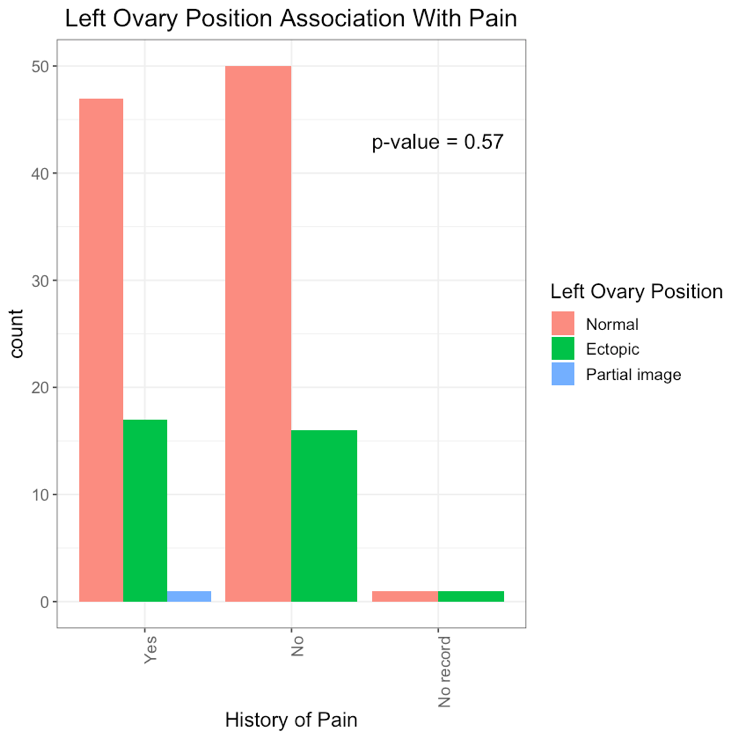

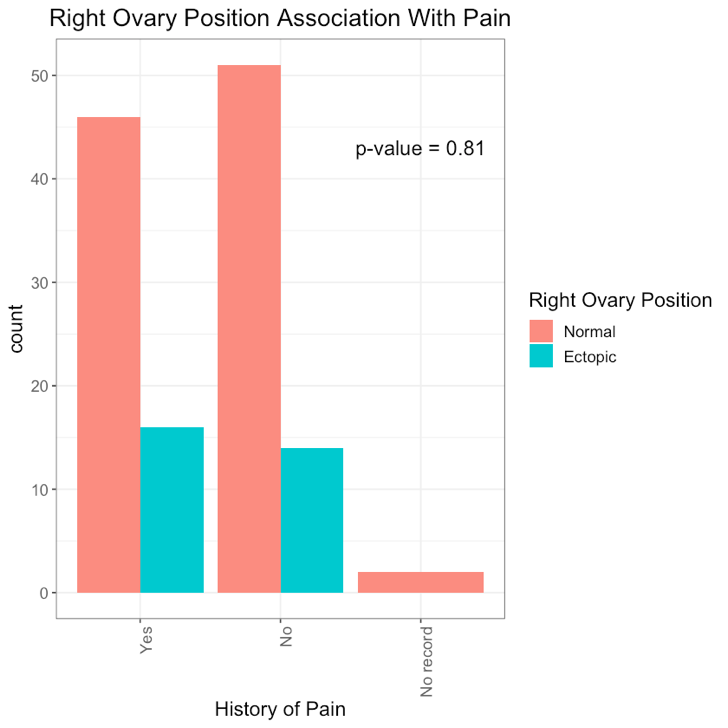


d

c

b

a

Figure S2: Association of pain history and a) ovarian position and b) presence of ovarian pathology


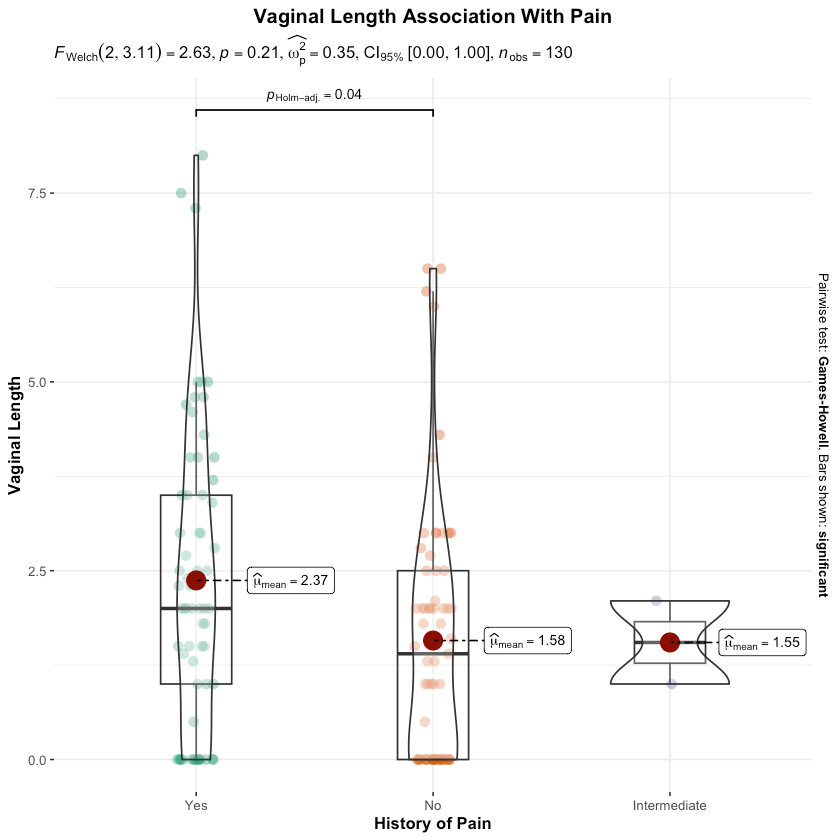


Figure S3: Association between vaginal length and history of pain
