# Supplementary material for: Magnetic resonance imaging and clinical features of Mayer–Rokitansky–Küster–Hauser syndrome: A 10‐year review from a dedicated specialist centre
Source: BJOG. 2024 Aug 12;132(1):64–71. doi: 10.1111/1471-0528.17928 (PMC11612609; doi:10.1111/1471-0528.17928)
Supplement: Supplementary file 2 — Figure S4 [file BJO-132-64-s002.docx]

**Figure S4: Image series to illustrate findings in MRKH**

**a)** MRI features of functional remnants vs non-functional remnants

**b)** Example of a fibrous band (sagittal T2)

**c** Example of relationship between anlage and ipsilateral ovary (Sagittal T2)

__

**d** Example of ectopic ovarian position (Axial T2 vs Axial SPAIR)

**e** example of ectopic renal position (Axial T2)

**f** Example of gynaecological pathology in anlage: fibroids

**g** Example of gynaecological pathology in anlage: endometrioma


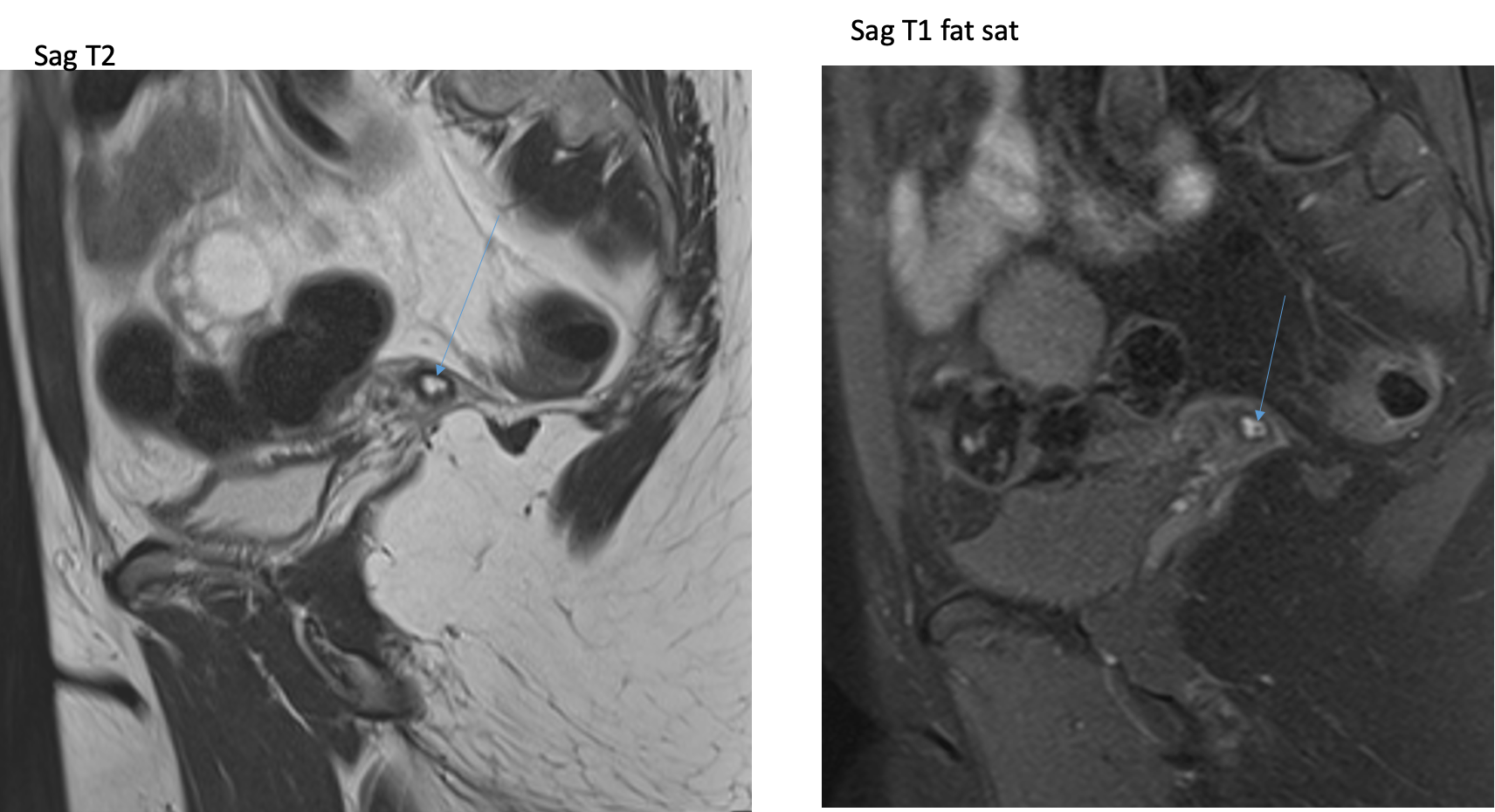


Within the left hemipelvis lying in the band of fibrous tissue connecting the two uterine remnants, there is a cystic area with a thick, low T1/T2 signal intensity rim that measures 6 x 8 x 13 mm and has internal content of high T2 and high non fatty T1 signal intensity. Appearances are suggestive of a small focal blood containing lesion with a haemosiderin rim in keeping with a small endometrioma.
