# Supplementary material for: Magnetic resonance imaging and clinical features of Mayer–Rokitansky–Küster–Hauser syndrome: A 10‐year review from a dedicated specialist centre
Source: BJOG. 2024 Aug 12;132(1):64–71. doi: 10.1111/1471-0528.17928 (PMC11612609; doi:10.1111/1471-0528.17928)
Supplement: Supplementary file 3 — Tables S1–S3 [file BJO-132-64-s001.docx]

Table S1:

| **Sequence** | **Scan time** | **FOV**  **(mm)** | **Slice thickness (mm)** | **Dist. Factor (GAP)** | **Slices** | **TR (ms)** | **TE (ms)** | **Averages** |
| --- | --- | --- | --- | --- | --- | --- | --- | --- |
| Axial T2W-TSE | 3:34 | 200 | 5 | 20 | 35 | 5740 | 87 | 2 |
| Sagittal T2W-TSE | 2:27 | 230 | 4 | 20 | 25 | 3930 | 82 | 2 |
| Axial T1W-TSE | 3:30 | 220 | 5 | 20 | 35 | 648 | 19 | 2 |
| Axial T2W-TSE (SFOV) | 2:51 | 180 | 3.5 | 10 | 23 | 4590 | 92 | 3 |
| Coronal T2W-TSE | 2:51 | 180 | 3.5 | 10 | 23 | 4590 | 92 | 3 |
| Sagittal T1W-TSE-FS | 4:35 | 220 | 4 | 25 | 20 | 617 | 19 | 2 |

***Table S1:*** Details of the parameters and sequences used to image patients with congenital gynaecological malformations. FOV = field of view, TR = repetition time, TE = echo time, T2W = T2 weighted, TSE = turbo-spin echo, SFOV = small field of view, FS = fat saturated.

***Table S2:*** Position of ectopically sited ovaries in women with MRKH

| Ectopic Ovary Position | Right | | % total  (n = 134) |
| --- | --- | --- | --- |
|  | n | % |  |
| Retrocaecal | 5 | 16.7 | 3.7 |
| High in pelvis | 5 | 16.7 | 3.7 |
| Overlying psoas | 4 | 13.3 | 3.0 |
| Overlying external iliac vessels | 3 | 10 | 2.2 |
| Lateral to ascending colon | 2 | 6.7 | 1.5 |
| Flank | 2 | 6.7 | 1.5 |
| Lateral to iliac vessels | 2 | 6.7 | 1.5 |
| Right upper quadrant | 1 | 3.3 | 0.7 |
| Left iliac fossa | 1 | 3.3 | 0.7 |
| Overlying iliacus | 1 | 3.3 | 0.7 |
| Right iliac fossa | 1 | 3.3 | 0.7 |
| Posterior to iliac vessels | 1 | 3.3 | 0.7 |
| Paracolic | 1 | 3.3 | 0.7 |
| Medial to anlage | 1 | 3.3 | 0.7 |
| Total | **30** | **100.0** | **22.4** |

| Ectopic Ovary Position | Left | | % total  (n = 134) |
| --- | --- | --- | --- |
|  | n | % |  |
| Overlying psoas | 10 | 27.8 | 7.5 |
| High in pelvis | 6 | 16.7 | 4.5 |
| LIF | 4 | 11.1 | 3.0 |
| Posterior to descending colon | 3 | 8.3 | 2.2 |
| Lateral to iliac vessels | 3 | 8.3 | 2.2 |
| Overlying external iliac vessels | 2 | 5.6 | 1.5 |
| Lateral to descending colon | 2 | 5.6 | 1.5 |
| Flank | 2 | 5.6 | 1.5 |
| Paracolic | 2 | 5.6 | 1.5 |
| Inguinal canal | 2 | 5.6 | 1.5 |
| Total | **36** | **100.0** | **26.9** |

Table S3: Type of renal and skeletal anomalies

| Skeletal Anomaly | Total | | % overall (n = 134) |
| --- | --- | --- | --- |
|  | **n** | **%** |  |
| Sacrum and coccyx | 6 | 33.3 | 4.5 |
| Multiple | 4 | 22.2 | 3 |
| Sacrum | 3 | 16.7 | 2.2 |
| Scoliosis | 2 | 11.1 | 1.5 |
| Coccyx | 1 | 5.6 | 0.7 |
| Hip | 1 | 5.6 | 0.7 |
| Lumbar spine | 1 | 5.6 | 0.7 |
|  | 18 | 100 | 13.4 |

| Renal Anomaly | Total | | % overall (n = 134) |
| --- | --- | --- | --- |
|  | **n** | **%** |  |
| Absent (right) | 10 | 27.8 | 7.5 |
| Absent (left) | 7 | 19.4 | 5.2 |
| Crossfused ectopia | 4 | 11.1 | 3 |
| Single pelvic kidney | 4 | 11.1 | 3 |
| Abnormal position - right pelvic kidney | 3 | 8.3 | 2.2 |
| Abnormal position - left pelvic kidney | 3 | 8.3 | 2.2 |
| Other abnormal position | 3 | 8.3 | 2.2 |
| Not classified | 2 | 5.6 | 1.5 |
|  | 36 | 100.0 | 26.9 |
